# In Silico Study of the Interactions of Anle138b Isomer, an Inhibitor of Amyloid Aggregation, with Partner Proteins

**DOI:** 10.3390/ijms232416096

**Published:** 2022-12-17

**Authors:** Maxim S. Kondratyev, Vladimir R. Rudnev, Kirill S. Nikolsky, Denis V. Petrovsky, Liudmila I. Kulikova, Kristina A. Malsagova, Alexander A. Stepanov, Arthur T. Kopylov, Anna L. Kaysheva

**Affiliations:** 1 Institute of Cell Biophysics, Russian Academy of Sciences, 142290 Pushchino, Russia; 2 Biobanking Group, Branch of Institute of Biomedical Chemistry “Scientific and Education Center”, 109028 Moscow, Russia

**Keywords:** alpha-synuclein, cyclophilin A, Anle138b isomer, molecular docking

## Abstract

Herein, we aimed to highlight current “gaps” in the understanding of the potential interactions between the Anle138b isomer ligand, a promising agent for clinical research, and the intrinsically disordered alpha-synuclein protein. The presence of extensive unstructured areas in alpha-synuclein determines its existence in the cell of partner proteins, including the cyclophilin A chaperone, which prevents the aggregation of alpha-synuclein molecules that are destructive to cell life. Using flexible and cascaded molecular docking techniques, we aimed to expand our understanding of the molecular architecture of the protein complex between alpha-synuclein, cyclophilin A and the Anle138b isomer ligand. We demonstrated the possibility of intricate complex formation under cellular conditions and revealed that the main interactions that stabilize the complex are hydrophobic and involve hydrogen.

## 1. Introduction

Amyloidosis is a heterogeneous group of diseases characterized by the deposition of amyloid fibrils in the extracellular matrix of tissues and organs [1,2]. In clinical practice, nosologies accompanied by amyloid formation are often named after fibrillar proteins (atrial amyloidosis, prolactinomas, synucleinopathies, etc.) [1,2]. Approximately 95% of amyloids consist of fibrillar proteins, and the remaining 5% comprise the P-component of serum amyloid glycoproteins [3]. Insoluble forms of fibrillar proteins are formed via the misfolding of the soluble form of the precursor proteins. To date, more than 30 proteins have been discovered to form human amyloid fibrils [4], 18 of which cause systemic amyloidosis and 22 are localized forms. Systemic amyloidosis develops due to the aggregation of light (AL) and heavy (AH) chains of immunoglobulin procalcitonin (ACal), which are α-synuclein (AαSyn), islet amyloid polypeptide C (AIAPP), prolactin (APro), Aβ protein precursor (Aβ), and lung surfactant protein (ASPCd) [4].

Aggregates of amyloid proteins are assumed to have a wide range of negative effects on the human body, including cytotoxic effects and tissue damage. Diseases characterized by the formation of amyloid or other aggregates of amyloid proteins (cytotoxic oligomers, protofibrils) can be divided into a group of neurodegenerative diseases, which include Alzheimer’s disease (amyloid protein: Aβ), Parkinson’s disease (PD; amyloid protein: AαSyn), and type 2 diabetes (amyloid AIAPP protein).

PD is a complex neurodegenerative disease characterized by bradykinesia, resting tremors, muscle rigidity, and postural instability. The pathogenesis of PD is accompanied by the loss of dopaminergic neurons in the substantia nigra and the presence of Lewy bodies (intraneuronal accumulation of aggregated AαSyn molecules) in neurons in various regions of the brain [5,6,7,8]. Owing to the key role of AαSyn aggregation in PD pathogenesis, AαSyn is an attractive target for potential disease-modifying therapies. However, the “many-sidedness” [9] of the protein due to (a) the disordered nature of AαSyn (random coil in aqueous solution, α-helical structure upon interaction with lipid membranes) [8,10,11]; (b) a complex aggregation mechanism: the nucleation-polymerization and nucleation-conversion-polymerization model [12,13,14,15]; and (c) the lack of structural information on AαSyn aggregation complicates the discovery of α-synamyloid blockers [9,16].

Turning to evidence-based data, there are low-molecular-weight compounds that block AαSyn misfolding and aggregation and, in a sense, promote the dissociation of aggregates [9,16]. Aggregation inhibitors of AαSyn molecules include chemical chaperones, ligands Anle138b [17,18,19], SynuClean-D [20,21], and peptidomimetics (NPT100-18a and NPT200-11) [22,23,24], which are at different stages of clinical trials [8,16,25].

In the present study, we aimed to focus on the insufficient knowledge about the structure and, consequently, diversity of the AαSyn protein. Ultimately, we expanded our understanding of the molecular architecture for the potential formation of protein complexes under cellular conditions involving the intrinsically disordered protein (IDP), AαSyn, and the globular protein, cyclophylin A (CypA). These proteins were selected as targets for flexible docking owing to a lack of knowledge regarding the binding process of the Anle138b isomer ligand to partner proteins. First, we evaluated the interactions that stabilized the Anle138b isomer ligand complex with the alpha-synuclein fragment. Thereafter, we analyzed the ternary complex, AαSyn, with the cellular partner protein CypA, and the target ligand as the model closest to physiological (cellular) conditions and assessed the potential binding of the Anle138b isomer ligand to the CypA partner protein in the monomeric and dimeric forms by all-atom flexible docking. Finally, we analyzed the results of cascade docking (in the presence of an excess of ligand molecules) for AαSyn and CypA protein complexes of different compositions. Molecular dynamics experiments and evaluation of binding energy components showed good agreement with the results of molecular docking for the most significant amino acids involved in the stabilization of the studied complexes.

## 2. Results

### 2.1. Characterization of the AαSyn–Anle138b Isomer Complex

**Figure 1:**
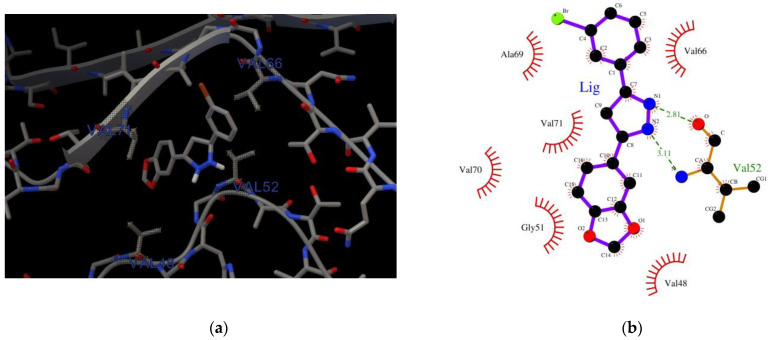

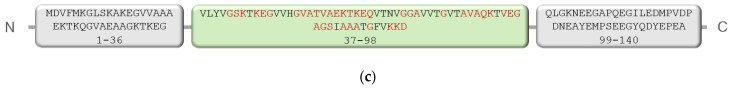
(**a**) Binding of the Anle138b isomer molecule to a fragment of the alpha-synuclein monomer (PDB ID 7V4C); (**b**) chart of interactions when Anle138b isomer binds to a fragment of the alpha-synuclein monomer (region: 37–98); (**c**) the amino acid sequence of AαSyn, the green box represents the region of the Val37–Asp98 sequence for which the molecular docking experiment was performed (PDB ID 7V4C), amino acid residues that belong to the disorder-promoting set are marked in red.

The structure of the AαSyn target was solved using electron microscopy with a resolution of 3.30 Å. The length of the amino acid sequence was 61 residues (Val37–Asp98), and the intact form of AαSyn was represented by 140 residues. The binding (affinity: −5.8 kcal/mol) of the Anle138b isomer molecule to a fragment of the alpha-synuclein monomer (PDB ID: 7V4C) is illustrated in Figure 1a.

The Val37–Asp98 region of AαSyn is rich in disorder-promoting amino acid residues [26,27] (Figure 1). The protein itself is a prominent representative of internally disordered proteins, which are characterized by extensive disordered regions. Based on an analysis of the obtained interactions (LigPlot), the geometry of the complexes was identified to be mainly stabilized by hydrophobic interactions with valine residues Val48, Val52, Val66, and Val71, and hydrogen bonding with Val52 of the main chain (Figure 1b).

**Figure 2:**
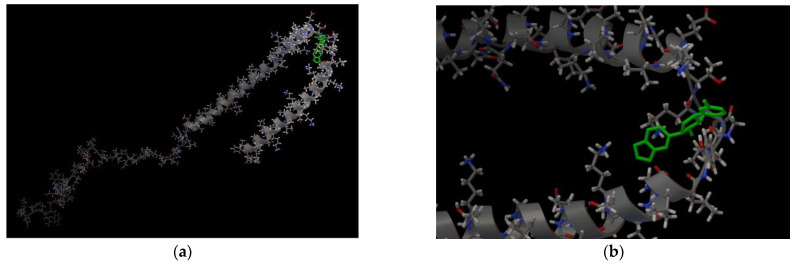

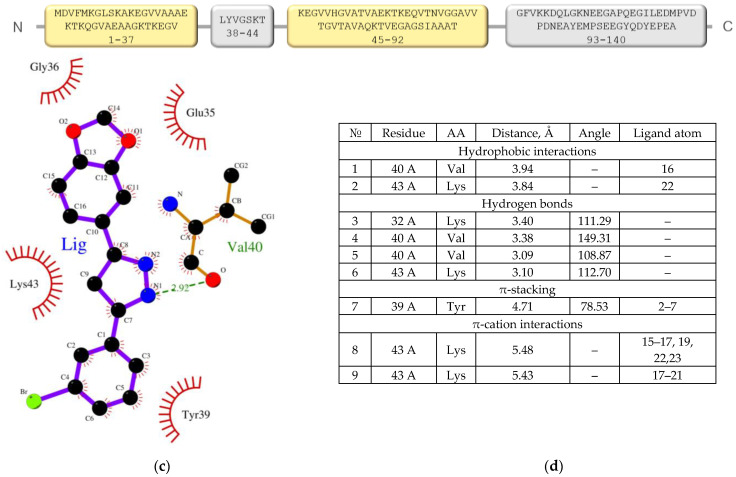
(**a**,**b**) Anle138b isomer molecule binding to full-length alpha-synuclein molecule (PDB ID 1XQ8). The 3D structure of the Anle138b isomer ligand is indicated in green. (**c**) Interactions diagram of Anle138b isomer with a fragment of the alpha-synuclein monomer. (**a**) full image size, (**b**) enlargement of the binding site image. The figure above shows the amino acid sequence of AαSyn, the blocks reflect the organization of secondary structure elements: yellow blocks correspond to alpha helices, gray blocks correspond to unstructured regions (coil). (**d**) Interaction options for Anle138b isomer binding to the alpha-synuclein monomer (PDB ID 1XQ8).

If the target for the Anle138b isomer ligand is the complete AαSyn structure in the amino acid sequence (140 amino acid residues, PDB ID 1XQ8), the amino acids responsible for this binding were also identified (affinity: −6.7 kcal/mol). The results are shown in Figure 2.

The AαSyn molecule (PDB ID 1XQ8) is characterized by two extensively structured regions and two alpha helices, Met1–Val37 and Lys45–Thr92 (Figure 2). Alpha helices form a supersecondary α-hairpin structure which is stabilized by hydrogen and hydrophobic interactions [28]. A short Leu38–Thr44 constriction exists between the alpha helices. According to the molecular docking data, the amino acid residues of this unstructured region are involved in the stabilization of the ligand/target complex. In this case, the condensed hydrophobic rings of the ligand are oriented inside the hydrophobic core of the α-hairpin. Of note, the AαSyn structure retained a large C-terminal unstructured tail in the Gly93-Ala140 region.

Owing to an analysis of the microenvironment of the bound ligand molecule in the Leu38–Thr44 region of the target protein, Val40, Lys43, Lys32, and Tyr39 were identified as the main partner residues for stabilizing the spatial position of Anle138b isomer. Of note, several different interactions were detected for the Lys43 residue: hydrophobic, hydrogen bonding, and π-cationic interactions, enabling the identification of the Lys43 amino acid residue as a key partner for binding to the Anle138b isomer ligand (Figure 2d). Attention should be paid to the rather small distances of bonds between the ligand and target for the identified types of interactions, which indicates the fundamental possibility of the formation of such complexes in the cell. However, data from the literature indicate the formation of the Anle138b isomer complex with different sizes of AαSyn aggregates, but not with a monomeric form [16].

### 2.2. Characterization of the AαSyn–CypA and Anle138b Isomer Complex

**Figure 3:**
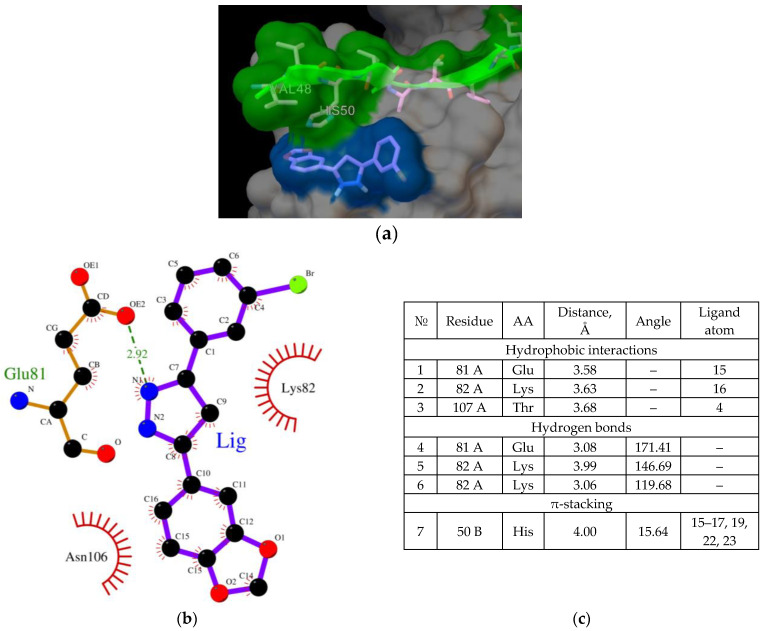
(**a**) Binding of the Anle138b isomer molecule to the AαSyn–CypA complex (PDB ID 6I42), a fragment of the surface of the AαSyn molecule is indicated in green, the surface of the CypA globular protein is indicated in gray, the molecular surface of the Anle138b isomer ligand is indicated in blue; (**b**) chart of interactions upon binding of Anle138b isomer to the AαSyn–CypA complex; (**c**) interactions diagram of Anle138b isomer with AαSyn–CypA complex.

In cells, AαSyn molecules form complexes with CypA [29,30]. CypA belongs to the family of immunophilins or chaperones containing the peptidyl prolyl cis/trans isomerase domain (PPIase). CypA is involved in basic neuronal functions, such as axonal transport and synaptic vesicle assembly; however, its most interesting role is neuroprotection. CypA prevents protein aggregation and binds unstructured or irregularly organized proteins [30]. As mentioned before, AαSyn is an unstructured protein, and its cellular partners can be different proteins and ligands. To study the binding features of the Anle138b isomer ligand and its partner protein, the experimentally known complex, AαSyn (region: Val48-Lys60), with the globular partner protein, CypA (PDB ID 6I42), as a target must be considered. The structure was resolved using X-ray crystallography with a resolution of 1.38 Å [29]. The results for the molecular docking of the ternary ligand-protein–protein complex are shown in Figure 3 (affinity: −7.1 kcal/mol).

As shown in Figure 3, the ligand was located on the mutual surface of AαSyn–CypA partner proteins, forming non-covalent interactions with each of the partner proteins. The binding site of the ligand with AαSyn also shifted toward the C-terminus of the protein, namely the Leu38–Thr44 region for the ligand–AαSyn complex compared to the Val48–Val52 region for the ligand–AαSyn–CypA complex. This phenomenon may be due to the experimental AαSyn–CypA structure, which was obtained for the AαSyn fragment. When interactions that stabilize the Anle138b isomer ternary complex and the AαSyn fragment with CypA are characterized, the hydrophobic interactions (His50, Asn106, Thr107) and hydrogen bonding (Glu81, Lys82) should be noted. The histidine residue belongs to alpha-synuclein, and the chart of interactions reveals its participation in π-stacking (Figure 3c). Based on our data, AαSyn in the ternary complex plays an important role in ligand binding. Currently, the map of interactions of the AαSyn–CypA protein complex in the absence of a ligand is well studied [29] and is described in more detail in the Section 3.

### 2.3. Molecular Docking: Characterization of the CypA and Anle138b Isomer Complex

**Figure 4:**
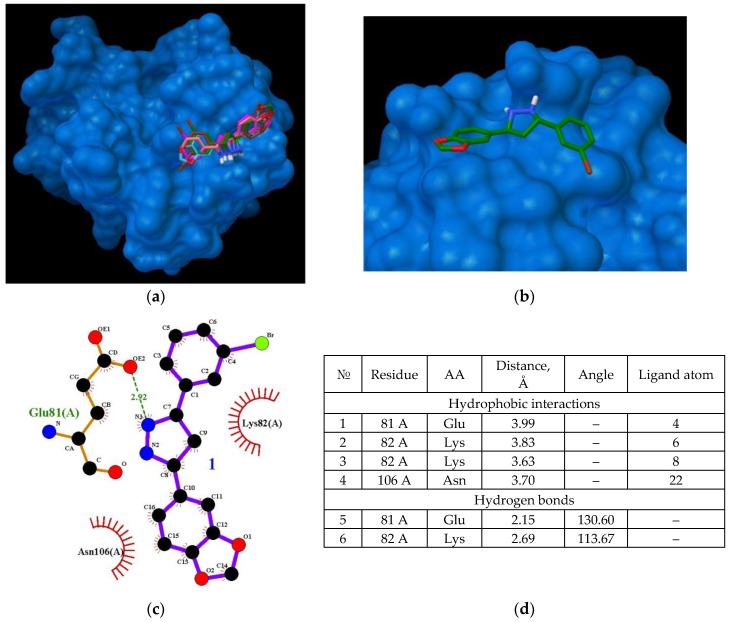
Binding of the Anle138b isomer molecule to CypA (a cellular partner protein of alpha-synuclein) (**a**) full size image; (**b**) an enlarged image of a possible CypA binding site; (**c**) interactions diagram of Anle138b isomer with cyclophilin; (**d**) interaction options for Anle138b isomer binding to CypA.

During AαSyn–CypA complex formation under physiological conditions, the potential role of CypA in the binding of the studied ligand must be examined. To determine whether the evaluated Anle138b isomer ligand can compete with the binding sites of the globular cellular partner protein (CypA) as a target (PDB ID 6I42) instead of binding to alpha-synuclein, all-atom flexible docking was performed (affinity: −7.3 kcal/mol). The results are shown in Figure 4.

Owing to the chart of this complex, the amino acid residues responsible for its stabilization could be determined (Figure 4d). The main residues that determine the binding of the Anle138b isomer ligand to cyclophilin include Glu81 and Lys82, which are involved in both hydrophobic interactions (part of the side chains) and hydrogen bonding (end groups). Notably, distances of the revealed probable hydrophobic and hydrogen interactions are quite small. Accordingly, CypA can also act as a target for the ligand by stabilizing the ligand molecule on the surface of the protein globule. Similar to the case of the Anle138b isomer–AαSyn–CypA ternary complex, the same amino acid residues were involved in the stabilization of the Anle138b isomer–CypA complex. Further, the binding site remained unchanged.

**Figure 5:**
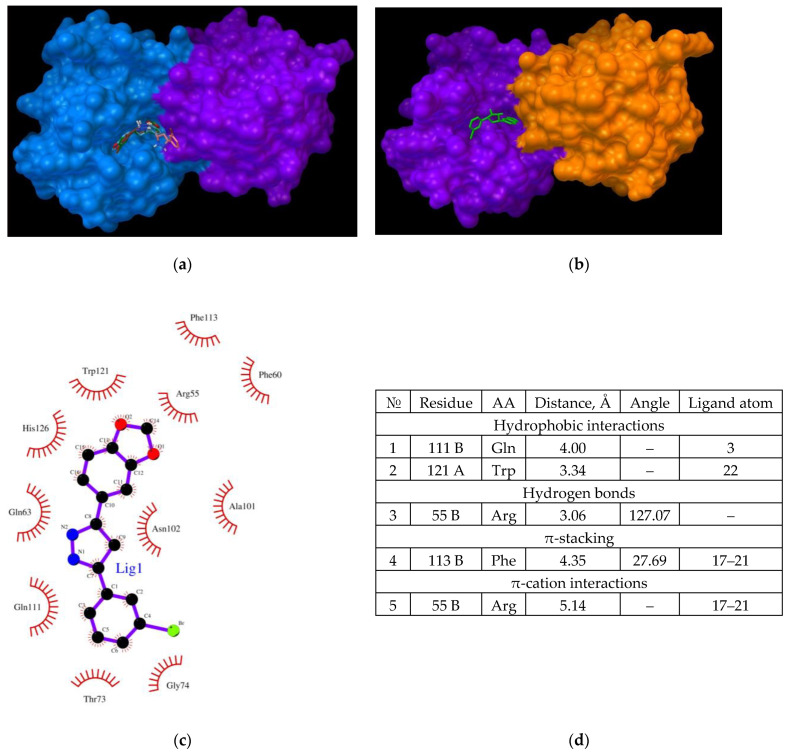
(**a**,**b**) binding of the Anle138b isomer molecule to the cyclophilin dimer (two views); (**c**) interactions diagram of Anle138b isomer with cyclophilin dimer; (**d**) interaction options for Anle138b isomer binding to the CypA dimer.

Recent studies by Lutomski and Zhang have provided information on the formation of CypA dimers and trimers [31,32]. However, the biological significance of this phenomenon has not been evaluated. Aligning with this data, the binding of Anle138b isomer to the CypA dimer proved interesting. This dimer was obtained using ZDOCK (https://zdock.umassmed.edu/), (accessed on 25 November 2022). The results are shown in Figure 5 (affinity: −7.5 kcal/mol).

**Figure 6:**
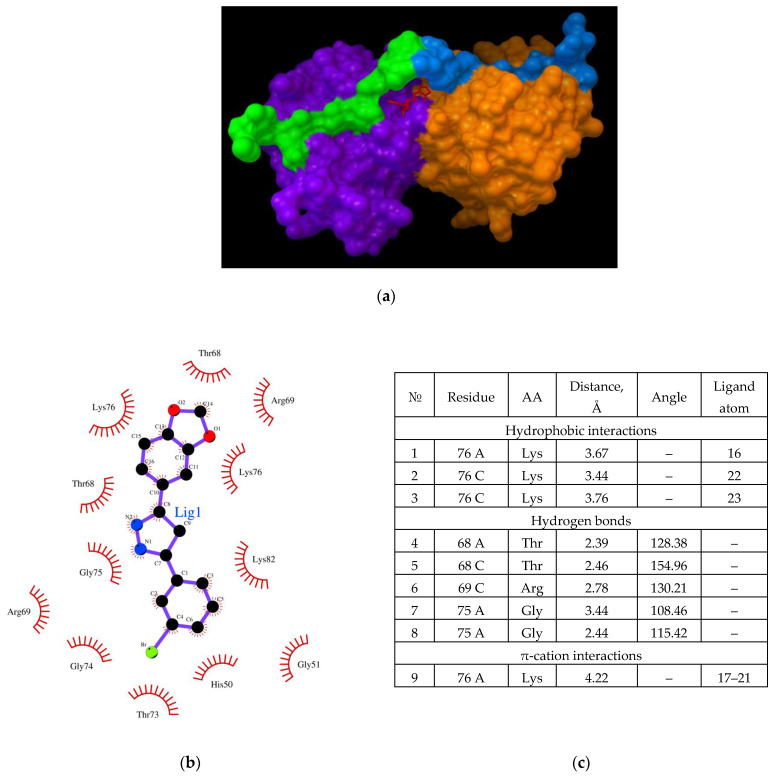
(**a**) Binding of the Anle138b isomer molecule to the dimer of the CypA complex (PDB ID 6I42). CypA subunits are shown in purple and orange, the AαSyn fragment is shown in green and blue, the ligand structure is shown in red; (**b**) chart of interactions upon binding of Anle138b isomer to the dimer of the CypA and AαSyn complex; (**c**) interactions diagram of Anle138b isomer binding with CypA dimer.

The location of ligand binding was found to differ from that of the monomer (Figure 5a,b). In the dimer, the ligand binds to the cleft surface formed by Arg55 (hydrogen bonding and π-cation interaction), Phe113 (π-stacking), and hydrophobic interactions through the Gln111 and Trp121 residues (Figure 5d). The chart of the bonds that stabilize the complex is illustrated in Figure 6c. Thus, for the formation of a CypA dimer, the ligand was located in the hollow formed by the interaction of the two CypA molecules.

To further predict the possible interaction of the Anle138b isomer ligand with cellular proteins, a molecular docking experiment was conducted for a more intricate complex by employing the dimeric form of the known AαSyn/CypA (PDB ID 6I42) complex. The dimer was obtained using ZDOCK, and the results obtained for the binding to Anle138b isomer are shown in Figure 6 (affinity: −7.7 kcal/mol).

In this version of the analysis, a different position was identified for the most favorable binding site compared to the dimer without the AαSyn fragment. It was found that the Anle138b isomer molecule binds on the surface of the globule in the groove under the chains of the AαSyn residues. Based on an analysis of the chart of the complex, the residues responsible for such binding could be identified (Figure 6b). The binding of the ligand occurs in a hollow formed by the surfaces of two cyclophilin globules, covered from above by the alpha-synuclein chains; the main residues stabilizing the complex are the Lys76 chains A and C (hydrophobic interactions through long chains and π-cationic interactions), and Thr68 residues (both chains, A and C). Further, Gly75A and Arg69C are involved in the formation of hydrogen bonds (Figure 6b). Simultaneously, the AαSyn fragment is directly involved in the formation of the binding site.

When the binding of a ligand to a molecule is assessed, the interactions that occur with an excess ligand must be analyzed. Such analysis is provided by cascade docking, in which a ligand is added in a step-by-step manner, molecule by molecule, after the optimal position of the previous ligand is incorporated into the target (see the Section 4). This approach allows full-atomic modeling of the binding of many ligand molecules and the detection of amino acid residues in the protein globule that can stabilize the formed complexes. Such calculations were performed for experimentally known molecules: series 1, the AαSyn fragment (PDB ID 7V4C); series 2, the full form of AαSyn (140 amino acid residues, PDB ID 1XQ8); and series 3, the CypA complex–AαSyn fragment (PDB ID 6I42). Each structure was surrounded by five Anle138b isomer ligand molecules.

**Figure 7:**
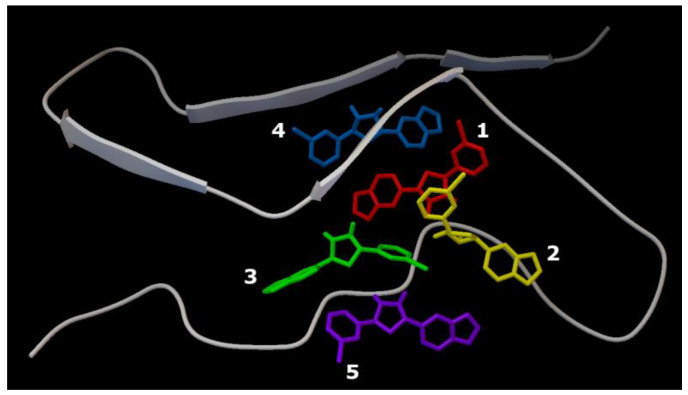
Five Anle138b isomer ligand binding sites on a filament of AαSyn (PDB ID 7V4C). Individual Anle138b isomer ligands are numbered: 1—red, 2—yellow, 3—green, 4—blue, 5—violet.

Series 1. Results of cascade docking for a short AαSyn fragment are shown in Figure 7.

The close localization of binding sites in the target protein should be noted (region: His50 andThr72). The ligand molecules were oriented close to parallel stacking and were characterized by similar energy characteristics, −5.7 kcal/mol to −6.9 kcal/mol (Table 1).

The sweeps for this type of interaction are shown in Table 1. It is demonstrated that structures are mainly stabilized via hydrophobic interactions with the residues, Val66 (Ligands 1 and 4), Val71 (Ligands 1 and 2), and Thr72 (Ligands 3 and 4), and hydrogen bonds with the residues, His50 (Ligands 1 and 2) and Val52 (Ligands 1 and 2).

**Figure 8:**
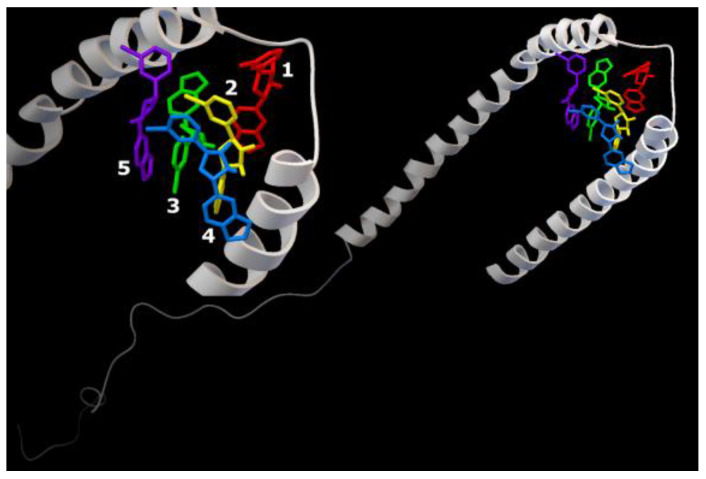
Five Anle138b isomer ligand binding sites on micelle-bound AαSyn (PDB ID 1XQ8). Individual Anle138b isomer ligands are as follows: 1—red, 2—yellow, 3—green, 4—blue, 5—violet.

Series 2. Results of cascade docking for AαSyn (140 amino acid residues) are shown in Figure 8.

The analysis of the stabilization features of complexes in the presence of five molecules of the Anle138b isomer ligand with a full-length AαSyn, as in series 1, revealed an extremely close localization of the binding sites, which is especially evident against the background of the extended structure of the molecule and its large molecular surface. The energy characteristics of the positions of the ligand calculated herein (and for the variant of cascade docking of the ligand molecules and the AαSyn fragment (series 1) range from −6.7 kcal/mol to −5.8 kcal/mol (Table 2).

The sweeps for this type of interaction are shown in Figure 8. Consequently, the structures were stabilized by hydrophobic interactions, hydrogen bonds, π-stacking, and π-cation interactions. The most significant hydrophobic interactions in the ligand–target complex include Lys43 (ligands 1 and 3), Val48 (ligands 3 and 4), and hydrogen interactions with the Lys32 residue (ligands 1–3). Of note, the fourth ligand was only bound to ligands 2 and 3, without contact with the protein chain, forming a second (stacking) layer of Anle138b isomer ligands (Table 2).

**Figure 9:**
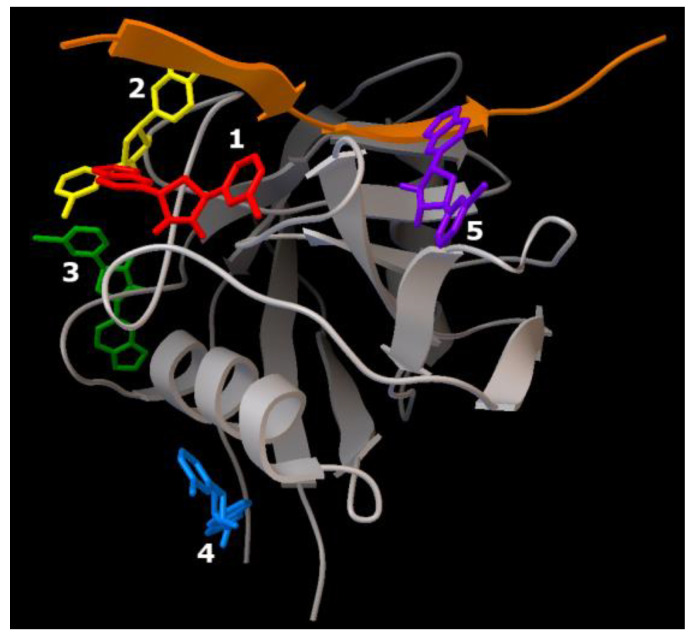
Five binding sites of the Anle138b isomer ligand of the complex of the AαSyn fragment and cyclophilin (PDB ID 6I42). Individual Anle138b isomer ligands have the following: 1—red, 2—yellow, 3—green, 4—blue, 5—violet.

Series 3. At the final stage of the work, cascade docking was carried out for the experimentally known (PDB ID 6I42) complex of the AαSyn fragment and its partner protein, CypA, as shown in Figure 9.

As this AαSyn–CypA target largely consists of a partner protein globule (and only a short peptide strand of AαSyn), the distribution of binding sites for all five Anle138b isomer ligands over the surface of the globule was expected. Only ligands 1 and 2 were found at the peptide interface. The energy characteristics of the five positions of the ligand calculated herein range from −7.1 kcal/mol to −6.6 kcal/mol (Table 3).

The sweeps for these interactions are shown in Figure 9. It was found that structures were stabilized by hydrophobic interactions, hydrogen bonds, π-stacking, and π-cationic interactions. For ligand 2, a halogen bond was found due to the presence of a bromine atom in the structure of the Anle138b isomer molecule.

In summary, the generally selective nature of the binding of Anle138b isomer ligand to alpha-synuclein molecules in different forms could be observed. For AαSyn complexes with the CypA partner protein, the preferred binding site is the interface between the two proteins. This study sought to assess the interactions of the ligand, whose interaction with AαSyn was postulated. Further, other ligands with such activity should be examined.

### 2.4. Molecular Dinamics: Characterization of the CypA and Anle138b Isomer Complex

Molecular dynamics experiments (GROMACS) were performed to determine the binding energies for the studied target–ligand complexes. The cumulative evaluation of the binding energies and the distribution of its components: van der Waal energy, electrostatic energy, polar solvation energy, and solvent-accessible surface energy (SASA energy) are presented in Table 4.

**Figure 10:**
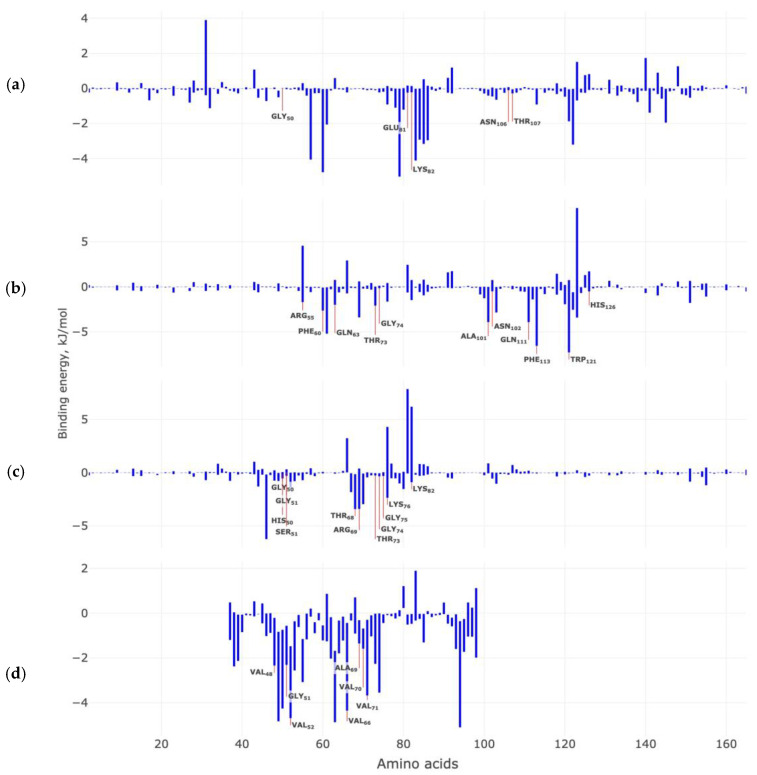
Contribution of target protein amino acids to the calculated binding energies (kJ/mol) with the Anle138b isomer ligand: (**a**) AαSyn–CypA; (**b**) CypA dimer; (**c**) dimer of the AαSyn–CypA complex; (**d**) AαSyn filament. In the figures, amino acids marked in yellow make a significant contribution to the stabilization of the complex according to the results of molecular docking experiments.

All energy components contribute significantly to the stabilization of the investigated variants of sets, with the exception of polar solvation energy. The calculated binding energy for ligand Anle138b isomer and AαSyn–CypA complex was −48.44 ± 26.11 kJ/mol, ligand Anle138b isomer and CypA dimer −98.53 ± 22.74 kJ/mol, ligand Anle138b isomer and dimer of the AαSyn–CypA complex −46.11 ± 24.69 kJ/mol, and ligand Anle138b isomer and AαSyn filament (homotrimer) −91± 27.29 kJ/mol. The time bases of the calculated binding energies are shown in Figure 10. The most stable in terms of cumulative binding energies are the ligand complexes with CypA dimer and AαSyn (filament).

Next, we calculated the contribution of each amino acid to the calculated values of the binding energy of each of the studied complex (Figure 10).

It was found that for the Anle138b isomer and AαSyn–CypA complex, the amino acid residues Ile57, Phe60, Met61, Trp121, Leu122, and Phe113 contribute most to the calculated binding energies (Figure 10a). At the same time, amino acids that were previously identified as significant in the stabilization of this complex in molecular docking experiments did not make a significant contribution to the calculated values of the binding energy.

For the second complex of Anle138b isomer and CypA dimer, we found that the most significant amino acid residues for the stabilization of the complex are the same as have been identified by the methods of molecular docking and molecular dynamics (Figure 10b). Such amino acid residues may include Arg55, Phe60, Gln63, Thr73, Gly74, Ala101, Gln111, Phe113 and Trp121. For the third Anle138b isomer complex and the dimer of the AαSyn–CypA, there is also agreement between the results of assessing the contribution of amino acid residues to complex formation: Thr68, Arg69, and Gly75 (Figure 10c). Additionally, for the fourth variant of Anle138b isomer and the AαSyn filament, we also observed the coincidence of the results of molecular docking and molecular dynamics for amino acid residues: Val48, Val52, Val66, Val71. For the studied complexes, the van der Waal energy, electrostatic energy, and polar solvation energy make the greatest contribution to the cumulative estimation of binding energies.

## 3. Discussion

In this study, we aimed to highlight some “gaps” in understanding the potential interactions of the Anle138b isomer ligand and the IDP, AαSyn. IDPs act as hubs in networks of protein interactions [33,34] and are important participants in cellular signaling mechanisms, including the regulation of transcription, translation, and the cell cycle [35,36]. The functional “many-sidedness” of IDPs, which is mediated by the formation of different types of protein complexes (protein–lipid raft, protein–protein, protein–ligand, protein–nucleic acid), is extremely important for studying the molecular mechanisms implemented in the cell in the state of “health” and “disease.”

The importance of studying the most probable variants of IDP interactions under physiological conditions was highlighted herein. These variants are not only closely associated with the implementation of normal cellular ontogenesis, but also with several nosologies. Our study focused on neurodegenerative diseases mediated by the formation of protein fibrils and aggregates (PD and AαSyn). Aligning with the above, the content of IDPs (25% of the total proteome) in the cell is strictly regulated to ensure accurate signaling in the cell cycle, while mutations in the protein-coding genes of IDPs or changes in the content of IDPs in the cell are associated with the development of diseases in humans [37,38,39]. Point mutation variants that cause PD have been annotated in the literature with high penetrance: Ala30Pro [40], Glu46Lys [41], Gly51Asp [42], His50Gln [43], Ala53Val [44], Ala53Glu [45], and Ala30Gly [46]. In some cases, carriers of point mutations in the AαSyn-coding gene are characterized by a relatively early onset of the disease (30–40 years), rapid progression, and severe cognitive impairment [47]. Of note, we revealed the participation (involvement) of these sites of amino acid substitutions in the formation of different variants of complexes between the ligand and alpha-synuclein molecule.

The presence of extensive unstructured regions in the AαSyn protein determines the presence of partner proteins in the cell, primarily chaperones, which are great representatives of agents that prevent the aggregation process that is destructive to cell life. Cellular globular chaperone peptidylprolyl isomerases (PPIases) catalyze the cis/trans proline isomerization of IDPs [48]. Cyclophilins are prominent representatives of PPIases and are highly represented in cellular proteins. CypA accounts for 0.1–0.6% of all cytosolic proteins [29]. Further, CypA plays an important role in the development of neurodegenerative diseases in humans. The role of CypA in neurodegenerative disorders is associated with the pathogenic aggregation of IDP [29], including the key pathogen in PD, AαSyn [49]. In a study by Favretto, two interaction sites between AαSyn and CypA were identified: Glu46–Gln62 in the central part of AαSyn and Val118–Glu131 in the proline-rich C-terminal region of AαSyn [29].

**Figure 11:**
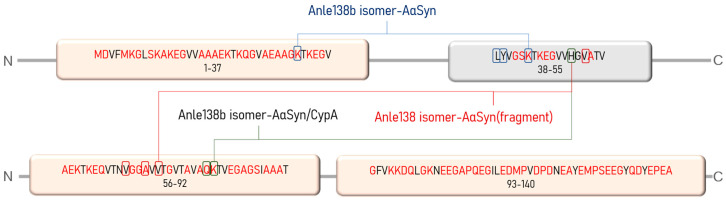
Amino acid sequence of AαSyn. Disorder-promoting amino acid residues are marked in red; pink blocks reflect clusters rich in disorder-promoting residues. Rectangles mark amino acid residues involved in the stabilization of the Anle138b isomer–AαSyn and Anle138b isomer–AαSyn–CypA complexes (according to molecular docking data).

The structural plasticity and functional diversity of IDPs are “encoded” in the amino acid sequence. Francois-Xavier Theillet (2013) aptly defined such code as the “alphabet of intrinsic disorder” [26]. The sequences of IDPs are rich in amino acid residues that promote disorders, such as proline, aspartic acid, methionine, lysine, arginine, serine, glutamine, and glutamic acid, and contain few residues that promote “order,” namely cysteine, tryptophan, tyrosine, isoleucine, phenylalanine, valine, leucine, histidine, threonine, and asparagine [27]. This alphabet was applied in our study to describe the results of different docking options. Indeed, the AαSyn protein contains approximately 70% disorder-promoting residues (Figure 11), which are organized into at least three distinct clusters (regions: Met1–Val37, Ala56–Ala91, and Gly93–Ala140).

Herein, the possible interactions of the Anle138B isomer with AαSyn and CypA proteins in different versions of the complex was described to enrich the current understanding of processes that may occur in living cells. Using molecular docking methods and molecular dynamics, we examined the most important events, in our opinion, that will expand the current understanding of the pathogenesis of PD. Comparison of the results of molecular docking and molecular dynamics showed good convergence for the most significant amino acids involved in the stabilization of the studied complexes.

## 4. Materials and Methods

### 4.1. Objects of Study

**Figure 12:**
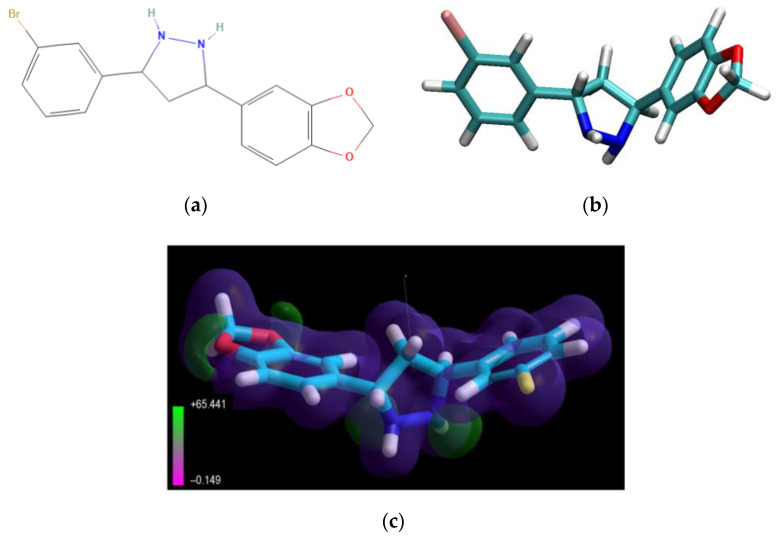
Structural formulas 2D (**a**) and 3D (**b**) of the Anle138b isomer; (**c**) electrostatic potential distribution for the Anle138b isomer molecule (according to the PM3 calculation, MOPAC 2021).

The low-molecular-weight drug, Anle138b isomer (Figure 12), is an oligomer modulator and aggregation inhibitor developed based on a systematic high-throughput screening campaign combined with medicinal chemistry optimization [25].

The value of the gas-phase heat of formation of the Anle138b isomer molecule is positive and amounts to 16.6 kcal/mol, which is typical for aromatic compounds (Figure 12). The Anle138b isomer ligand was detected after two consecutive screening rounds. Of note, the ligand does not interact with the AαSyn monomer, but binds to the hydrophobic pocket in the oligomeric states of the target protein, preventing the formation of the β-layer and AαSyn aggregates [16]. Anle138b isomer specifically inhibits the formation of AαSyn in oligomers in vitro and in the human cell line, HEK293 [50]. The physical and chemical properties of Anle138b isomer are outlined in Table 5.

AαSyn and CypA were selected as target proteins for the Anle138b isomer ligand (Table 6). AαSyn belongs to a large group of internally disordered proteins, while CypA is a cellular chaperone with a globular and compact structure.

### 4.2. Molecular Docking

The protein structures (as targets) were prepared for docking according to the standard scheme for the Autodock Vina package, and the atoms (together with their coordinates) of the solvent, buffer, and ligand molecules were removed from the PDB input file. Before the numerical calculations were performed, a charge was placed on the surface of the proteins using MGLTools. The center of the molecule and the parameters of the box («cells») were set manually, ensuring that the entire protease molecule was placed within the computational area of space.

We assembled the AαSyn–CypA dimer complex by rigid protein–protein docking using the Z-Dock service. This procedure has been repeatedly tested by various teams of researchers and is described in detail in the article [52].

The Anle138b isomer (ligand) structure model was drawn in the HyperChem molecular designer; this structure was consistently optimized in the AMBER force field, and then quantum-chemically in the PM3 (п**a**к**e**т MOPAC) [53]. The optimization consisted of calculating the complete electronic structure of the molecule, characterizing the geometry of all chemical bonds, and valence and torsion angles of the molecule. In the same calculation, the distribution of partial charges on the surface of the ligand and its dipole moment were determined. The ligands in the docking calculations had maximum conformational freedom. Of note, rotation of the functional groups around all single bonds was allowed. The charge arrangement on the Anle138b isomer molecule and its protonation–deprotonation was performed automatically in the MGLTools 1.5.6 package.

To obtain the in silico results, sequential (cascade, multiple) docking was applied. For Anle138b isomer, the five binding sites were successively modeled as described below. First, docking was performed for the «protein–ligand» complex. Thereafter, the model was augmented with another ligand (i.e., three-monomer chain) and so on. Thus, «blind» or cascade docking was employed. First, the optimal position of the ligand was calculated; the structure of the ligand (fixed at the docking site) became an integral part of the target. Therefore, this binding site (with a high affinity for the ligand) was blocked. The position of the second ligand, whose molecule (in its optimal position on the globule) also became a part of the target, was subsequently calculated. Ultimately, both bound ligands become part of the receptor. These iterative searches and filling of the optimal binding sites on the target surface were repeated until the positions of all five ligands were calculated. Of note, this cascade docking is reasonable for modeling ligand–receptor interactions for excess ligand molecules. Using this procedure, we successively excluded several possible binding sites of ligands via stepwise filling of the available areas on the surface of the protein–ligand complex. In the course of the analysis of the obtained complexes, the terms, «bonds» (understanding drawn interatomic chemical bonds, hydrogen bonds between their donors and acceptors) and «interactions» (mainly understanding hydrophobic bonds), were used. In general, cascade docking enables modeling and analysis of the binding between the protein and ligands in excess of ligands at the atomic level (Anle138b isomer).

The analysis of the docking results was nontrivial. To obtain the «sweeps» of the interactions, the LIGPLOT+ package [54] was employed. From the three-dimensional coordinates of the atoms of the protein–ligand complex, interaction diagrams depicting the schemes of hydrogen bonds and hydrophobic contacts between the ligand and elements of the main or side chain of the protein were generated. For in-depth analysis of the interactions between the constituent parts of the complexes, the PLIP (Protein–Ligand Interaction Profiler) package [55] was employed. They are distinguished by accuracy and content. The PLIP server can detect and display the following interactions: hydrogen bonds, hydrophobic interactions, water bridges, parallel stacking, perpendicular stacking, π-cation bonds, halogen bonds, salt bridges, and metal complexes. In addition to the figure, the package displays tables with distances, angles, and types of detected interactions, which are important for the analysis of previously obtained experimental data.

### 4.3. Molecular Dynamics

Molecular dynamics simulations were performed using GROMACS software packet. Ligand topology structure were obtained from the Automated Topology Builder (ATB) resource [56]. Calculations were performed using GROMOS547 forcefield modified by ATB. This forcefield was chosen because it supports bromine (contained in Anle138b isomer) and there was already a prepared topology for the ligand in the ATB service (ATB provides topology for GROMOS54a7 forcefield only). It is not as popular as AMBER Force Field but still used in MD experiments [57].

The molecular dynamics process was based on one described in GROMACS protein–ligand tutorial [58]. Simulations were run in cubic boxes. The box size was automatically calculated by GROMACS based on complex size and adding 1 nm from the complex size to the box border. The water SPC model was used as a solvent as it is supported by the chosen forcefield and grants significant simulation performance improvement with acceptable losses in accuracy. Systems were neutralized automatically with Na^+^ or Cl^−^ ions (one at a time). Then, energy minimization and equilibration steps were performed using the configuration found in the same tutorial [58]. The molecular dynamics themselves were performed for 100 ns in three different runs (using the same equilibrated complex topology for each run). The simulation system temperature was set to 300 K.

Analysis was performed for the binding energies of the protein–ligand complex trajectory following the MM/PBSA approach [59,60,61]. We performed analyses of the binding energy of protein–ligand complexes and binding energy distributions for every residue. This approach to trajectory analysis was based on one found in the study [62].

## 5. Conclusions

Pathophysiological conditions of neurodegenerative diseases are undoubtedly related to protein misfolding. The organization of such proteins into relatively ordered structures, such as fibrillar intracellular and extracellular amyloids, leads to tissue and brain damage. In these pathologies, the appearance of protein aggregates indicates inefficient cellular reactions involving molecular chaperones that contribute to the correct folding of partner proteins [30]. This study aimed to expand our understanding of the possible mechanisms of interaction of the Anle138b isomer ligand, which is promising for clinical use and prevents the aggregation of cellular amyloidogenic proteins. This field of study reflects the urgent clinical need to develop neuroprotective molecules targeting potentially novel molecular targets. Our understanding of the interaction of the Anle138b isomer ligand with variants of the alpha-synuclein molecule was expanded, and the possibility of forming more intricate complexes with CypA, a partner protein of alpha-synuclein, was revealed.

Our study paves the way for experimental confirmation, including an analysis of mutant forms of alpha-synuclein, which cause high disease penetrance, early onset, and rapid progression of PD.

## Figures and Tables

**Table 1:** Interactions of five Anle138b isomer ligand molecules with a filament of AαSyn (PDB ID 7V4C).

Interactions	Residue	AA	Distance, Å	Angle	Ligand Atom	Affinity (kcal/mol)
First ligand						
Hydrophobic	66A	Val	3.86	–	95	−5.8
	66A	Val	3.60	–	95	
	69 A	Ala	3.77	–	95	
	71 A	Val	3.61	–	115	
	71 A	Val	3.87	–	108	
Hydrogen bonds	50 A	His	3.35	121.47	–	
	52 A	Val	2.18	156.21	–	
	52 A	Val	1.84	157.17	–	
Second ligand						
Hydrophobic	53 A	Ala	3.58	–	84	−6.9
	55 A	Val	3.94	–	91	
	71 A	Val	3.63	–	75	
	71 A	Val	3.77	–	76	
Hydrogen bonds	50 A	His	2.50	113.40	–	
	52 A	Val	2.30	130.98	–	
	54 A	Thr	3.00	136.63	–	
Third ligand						
Hydrophobic	47 A	Gly	3.40	122.20	–	−6.3
	72 A	Thr	2.41	114.07	–	
	72 A	Thr	2.91	105.00	–	
Fourth ligand						
Hydrophobic	52 A	Val	3.48	–	45	−6.3
	66 A	Val	3.60	–	45	
	72 A	Thr	3.92	–	30	
Hydrogen bonds	70 A	Val	2.13	149.27	–	
Fifth ligand						
Hydrophobic	46 A	Glu	3.53	–	3	−5.7
	49 A	Val	3.64	–	23	
Hydrogen bonds	48 A	Val	2.11	163.86	–	
π-cation	50 A	His	4.16	–	17–21	

**Table 2:** Interactions of five Anle138b isomer ligand molecules with micelle-bound AαSyn (PDB ID 1XQ8).

Interactions	Residue	AA	Distance, Å	Angle	Ligand Atom	Affinity (kcal/mol)
First ligand						
Hydrophobic	40 A	Val	3.94	–	108	−6.7
	43 A	Lys	3.84	–	114	
Hydrogen bonds	32 A	Lys	3.40	111.29	–	
	40 A	Val	3.36	149.31	–	
	40 A	Val	2.61	108.87	–	
	43 A	Lys	2.56	112.70	–	
π-stacking	39 A	Tyr	4.71	78.53	94–99	
π-cation	43 A	Lys	5.48	–	107–109, 111, 114, 115	
	43 A	Lys	5.43	–	109–113	
Second ligand						
Hydrophobic	33 A	Thr	3.79	–	91	−5.8
Hydrogen bonds	32 A	Lys	2.47	138.24	–	
	36 A	Gly	2.51	128.51	–	
Third ligand						
Hydrophobic	43 A	Lys	3.96	–	62	−6.2
	48 A	Val	3.79	–	68	
	48 A	Val	3.96	–	62	
	52 A	Val	3.90	–	68	
Hydrogen bonds	32 A	Lys	2.37	129.57	–	
	52 A	Val	3.29	112.57	–	
Fourth ligand						
Hydrophobic	45 A	Lys	3.76	–	6	−5.5
	48 A	Val	3.55	–	6	
	49 A	Val	3.99	–	7	

**Table 3:** Interactions of five Anle138b isomer ligand molecules with complex AαSyn–CypA (PDB ID 6I42).

Interactions	Residue	AA	Distance, Å	Angle	Ligand Atom	Affinity (kcal/mol)
First ligand						
Hydrophobic	81 A	Glu	3.58	–	107	−7.1
	82 A	Lys	3.63	–	108	
	107 A	Thr	3.68	–	96	
Hydrogen bonds	81 A	Glu	2.07	171.41	–	
	82 A	Lys	3.13	146.9	–	
	82 A	Lys	2.43	119.68	–	
π-stacking	50 B	His	4.00	15.64	107–111, 114, 115	
Second ligand						
Hydrophobic	76 A	Lys	3.92	–	73	−7.0
	76 A	Lys	3.74	–	72	
Hydrogen bonds	68 A	Thr	2.33	128.37	–	
	75 A	Gly	3.49	108.72	–	
	75 A	Gly	2.54	109.73	–	
Halogen bonds	80 A	Gly	3.72	150.45	–	
Third ligand						
Hydrophobic	46 A	Phe	3.79	–	52	−7.0
	49 A	Lys	3.24	–	62	
	67 A	Phe	3.47	–	52	
Hydrogen bonds	47 A	Gly	2.94	115.96	–	
	47 A	Gly	2.73	111.32	–	
	49 A	Lys	2.52	129.52	–	
	49 A	Lys	2.94	108.80	–	
π-cation	49 A	Lys	4.74	–	63–97	
Fourth ligand						
Hydrophobic	26 A	Ala	3.74	–	45	−6.7
	31 A	Lys	3.44	–	625	
	79 A	Tyr	3.57	–	29	
π-cation	37 A	Arg	5.36	–	40–44	
Fifth ligand						
Hydrophobic	88 A	Phe	3.72	–	4	−6.6
	105 A	Pro	3.48	–	2	
	125 A	Lys	3.94	–	15	
	125 A	Lys	3.62	–	23	
	125 A	Lys	3.59	–	14	
Hydrogen bonds	85 A	Asp	3.18	121.95	–	
	102 A	Asn	3.26	127.71	–	
	104 A	Gly	3.06	154.03	–	

**Table 4:** Free interaction energies for studied ligand–target variant complexes (MM-PBSA analysis).

Ligand Anle138b and Target	Van der Waal Energy (kJ/mol)	Electrostatic Energy (kJ/mol)	Polar Solvation Energy (kJ/mol)	SASA Energy (kJ/mol)	Binding Energy (kJ/mol)
AαSyn–CypA complex (PDB ID 6I42)	−67.96 ± 34.5	−26.05 ± 29.6	53.2 ± 34.47	−7.7 ± 3.85	−48.44 ± 26.11
CypA dimer	−151.56 ± 18.9	−34.54 ± 13.6	102.59 ± 18.1	−15 ± 1.33	−98.53 ± 22.74
Dimer of the AαSyn–CypA complex	−91.36 ± 33.8	−16.58 ± 2.3	71.63 ± 10.2	−9.87 ± 3.36	−46.11 ± 24.69
AαSyn (filament, PDB ID 7V4C)	−140.93 ± 47.68	−14.66 ± 5	78.87 ± 28.95	−14.37 ± 3.32	−91 ± 27.29

**Table 5:** Physicochemical properties of the Anle138b isomer.

Ligand	IUPAC (Formula)	Class	Mw, Da	Donor HB *	Acceptor HB	SASA **, Å^2^	Dipole Moment, Debay	Sizes	Clinical Phase	IC50, nM ***	Ref.
Anle138b isomer	(3-(1,3-benzodioxol-5-yl)-5-(3-bromophenyl)-1H-pyrazolidine	pyrazolidine	347.21	2	4	313.74	1.61	13.514 Å 6.402 Å 4.381 Å	Preclinical	900	[51]

* hydrogen bond; ** solvent accessible surface area; *** H-SY5Y neuroblastoma cell model of alpha-synucleinopathy.

**Table 6:** Structures of AαSyn and CypA used for molecular docking with Anle138b isomer.

Target	PDB ID (WebLink)	Uniprot ID	Shape of Protein	Resolution, Å	Ligand (Locus, a.a.)	Method	Length, a.a.
AαSyn (micelle-bounded)	1XQ8 (https://www.rcsb.org/structure/1XQ8, accessed on 25 November 2022)	P37840	IDP *	–	–	NMR **	140
AαSyn (filament)	7V4C (https://www.rcsb.org/structure/7V4C, accessed on 25 November 2022)	P37840		3.30	–	EM ***	140 ****
CypA	6I42 (https://www.rcsb.org/structure/6I42, accessed on 25 November 2022)	P62937	Globular	1.38	AαSyn (1–13)	X-ray	164

IDP * intrinsically disordered protein; NMR ** nuclear magnetic resonance; EM *** electron microscopy; 140 **** homotrimer.

## Data Availability

Not applicable.
